# Response-shift effects in neuromyelitis optica spectrum disorder: a secondary analysis of clinical trial data

**DOI:** 10.1007/s11136-020-02707-y

**Published:** 2020-12-02

**Authors:** Carolyn E. Schwartz, Roland B. Stark, Brian D. Stucky

**Affiliations:** 1 DeltaQuest Foundation, Inc, 31 Mitchell Road, Concord, MA 01742 USA; 2 Departments of Medicine and Orthopaedic Surgery, Tufts University Medical School, Boston, MA USA

**Keywords:** Neuromyelitis optica spectrum disorder, Definitive neuromyelitis optica, Neurologic, Response shift, Clinical trial, Patient-reported outcome, Clinician-assessed outcome

## Abstract

**Background:**

Researchers have long posited that response-shift effects may obfuscate treatment effects. The present work investigated possible response-shift effects in a recent clinical trial testing a new treatment for Neuromyelitis Optica Spectrum Disorder (NMOSD). This pivotal trial provided impressive support for the drug Eculizumab in preventing relapse, but less strong or null results as the indicators became more subjective or evaluative. This pattern of results suggests that response-shift effects are present.

**Methods:**

This secondary analysis utilized data from a randomized, double-blind trial evaluating the impact of Eculizumab in preventing relapses in 143 people with NMOSD. Treatment arm and then relapse status were hypothesized ‘catalysts’ of response shift in two series of analyses. We devised a “de-constructed” version of Oort structural-equation modeling using random-effects modeling for use in small samples. This method begins by testing an omnibus response-shift hypothesis and then, pending a positive result, implements a series of random-effects models to elucidate specific response-shift effects.

**Results:**

In the omnibus test, the ‘standard quality-of-life (QOL) model’ captured substantially less well the experience of placebo as compared to Eculizumab group. Recalibration and reconceptualization response-shift effects were detected. Detected relapse-related response shifts included recalibration, reprioritization, and reconceptualization.

**Conclusions:**

Trial patients experienced response shifts related to treatment- and relapse-related experiences. Published trial results likely under-estimated Eculizumab vs. Placebo differences due to recalibration and reconceptualization, and relapse effects due to recalibration, reprioritization, and reconceptualization. This novel random-effects- model application builds on response-shift theory and provides a small-sample method for better estimating treatment effects in clinical trials.

**Electronic supplementary material:**

The online version of this article (10.1007/s11136-020-02707-y) contains supplementary material, which is available to authorized users.

## Introduction

Despite the advantages of rigorous clinical trial designs in providing unbiased estimates of treatment outcomes, these designs may also lead to somewhat paradoxical findings. For example, a treatment may have an unarguable benefit on objective outcomes but a less clear impact on more subjective outcomes. Research on response-shift effects provides a theory-driven and empirically testable path toward understanding such paradoxes. “Response shift” refers to the idea that when individuals experience a change in health status, they may change their internal standards, values, or conceptualization of a target construct like “quality of life” (QOL) [1, 2]. Over the past two decades, research in a broad range of therapeutic areas has supported that response-shift effects can influence clinical research findings, and can represent positive and negative adaptation [3–12]. While response-shift effects are generally small, they can influence study conclusions and are thus not inconsequential [3, 4].

The current methods for detecting response-shift effects [1, 13, 14] work with the idea that unexpected levels of QOL scores reflect adaptation [9, 11, 12, 15–20]. For example, if a clinician-assessed outcome does not agree with a patient-reported outcome (PRO), this “discrepancy” may signal patients’ changes in internal standards, values, or conceptualization of the target construct (e.g., QOL) [17]. Rather than suggesting that either the clinician-assessed or the patient-reported outcome is flawed or biased, this discrepancy suggests that there is ‘more than meets the eye,’ and that a deeper investigation of the situation is warranted. A recent study of people with spinal cord injury (SCI) reported that while objective measures of motor and cognitive function had stabilized one to five years post-injury, patient-reported outcomes reflected recalibration and reconceptualization response shifts [5]. Specifically, patients experienced improvements in physical functioning primarily by dint of improvements in physical role performance over time (recalibration) [5]. They also appeared to change their conceptualization of QOL over time such that over the long-term follow-up, the people with SCI stopped considering their SCI per se as part of their general health, and instead only considered SCI sequelae as part of their general health [5]. These response-shift effects may be important in understanding the full range of dynamics that matter for QOL, as in for example, measures of clinical significance in other patient populations such as with multiple sclerosis and spinal disorders [21, 22].

Researchers have long posited that response-shift effects may obfuscate treatment effects. A substantial number of articles have discussed the importance of response-shift effects in clinical trials (e.g., [23, 24]), and several studies have tested for response-shift effects in clinical trials [6, 11, 25–29]. Several of these studies used the then-test method, a method prone to recall bias and lack of specificity which challenges interpretation [30–32]. One of the studies used the relative-importance method to evaluate reprioritization response shifts [11], and two of these studies either combined the then-test with the Schedule for the Evaluation of Individualized QOL (SEIQOL) individualized method [27] or used another individualized metric, the Patient-Generated Index (PGI) [29]. These latter two studies thus provide a fuller, qualitative context to the respondents’ changes in priorities and conceptualizations of QOL. The metrics are, however, difficult to harness in quantitative metrics that can help to interpret trial outcomes in comparison to metrics that ignore response-shift effects.

The present work aimed to investigate possible response-shift effects in a recent clinical trial (*n* = 143) testing a new treatment for Neuromyelitis Optica Spectrum Disorder (NMOSD) [33]. This uncommon but severe form of demyelinating disease is a relapsing, autoimmune, inflammatory disorder that typically affects the optic nerves and spinal cord, leading to blindness and paralysis [34]. Often initially misdiagnosed as having multiple sclerosis, NMOSD patients face a frightening trajectory of severe relapses that leave residual neurologic disability and bring about unpredictable and disabling future attacks [35].

This NMOSD clinical trial provided impressive support in preventing relapse (primary outcome) for the drug Eculizumab. It also provided support for Eculizumab on the more objective secondary outcomes, which were clinician-assessed indicators as well as the EQ-5D utility measure of health state [36]. There was, however, less strong support as the indicators became more subjective or evaluative (e.g., EQ-5D visual analogue scale  or EQ-5D VAS indicator of global health; evaluative physical functioning), with null results for the evaluative self-report measure of mental functioning [37]. This pattern of results led us to hypothesize that response-shift effects are present.

Response-shift methods for detecting effects in secondary analyses often rely on relatively large sample sizes [38]. For example, the abovementioned SCI study used Oort’s Structural Equation Modeling (SEM) [10, 39], a well-vetted method that has been used in a number of secondary analyses of observational data [4, 10, 40–42]. This approach provides an ordered series of steps that test for response-shift effects, with later steps conducted only if earlier steps pass muster. The sample for the present study is too small for an Oort SEM analysis. Instead, we devised a “de-constructed” approach that is more appropriate for use with small samples. This method begins by testing an omnibus response-shift hypothesis and then implements a series of analyses aimed to elucidate what is uncovered in the omnibus test. We investigated possible effects first for Treatment Arm as a ‘catalyst’ of response shift due to the substantial health-state changes that differentiated the two groups [2, 17]. We then examined Relapse Group as a catalyst, to better understand these findings.

## Methods

### Sample and trial procedure

**Fig. 1:**
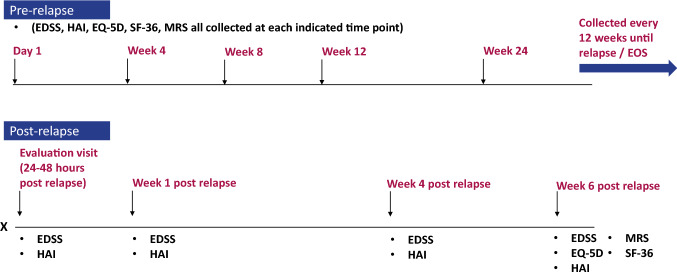
Timing of PRO measurement collection in the clinical trial design. This study schema provides the timing of clinician- and patient-reported outcome collection over the course of the trial

This secondary analysis utilized data from a randomized, double-blind, time-to-event trial evaluating the impact of Eculizumab in preventing relapses in 143 people with NMOSD. Eligible participants were patients of age 18 years or older, who had a diagnosis of NMOSD or neuromyelitis optica chronic medical condition. This international trial was recruited from 80 sites over four continents. Figure 1 provides the timing of clinician- and patient-reported outcome collection over the course of the trial. (For complete details on trial inclusion and exclusion criteria and procedures see reference [33]). The trial was conducted in accordance with the provision of the Declaration of Helsinki, the International Conference on Harmonization guidelines for Good Clinical Practice, and applicable regulatory requirements. The trial was approved by the institutional review board at each participating institution. All the patients provided written informed consent before participation.

### Measures

For the present analysis, we included information about treatment arm (i.e., Eculizumab vs. Placebo) as well as the following clinician- and patient-reported outcome data and information about relapse.

**Clinician-assessed outcomes.**Clinicians who were blind regarding trial-group assignment rated patients’ disability on the Kurtzke Expanded Disability Status Scale (EDSS) [43]. This standard neurological outcome tool assigns scores based on eight Kurtzke Functional Systems that include signs of disability (pyramidal, cerebellar, brainstem, sensory, bowel/bladder control, visual, cerebral, and other). The EDSS score ranges from 0 [no disability] to 10 [death]. Treating clinicians or appropriately trained staff members evaluated patients using the modified Rankin scale (MRS) [44], which assesses the degree of dependence in daily activities; scores range from 0 (no disability) to 6 (death). The Hauser Ambulation Index (HAI) [45] focuses on mobility disability by assessing how much time and degree of assistance is needed to walk 25 feet. Its scores range from 0 to 9, with higher scores indicating increased impairment.

**PROs.**Patients completed the European Quality of Life 5-Dimension 3-Level (EQ-5D-3L) questionnaire [36]. For the purpose of this study, we included the EQ-5D VAS item, a subjective global score of self-reported health ranging from 0 (worst imaginable health) to 100 (best imaginable health). The Short-Form-36v2 (SF-36v2™) [46] is a generic evaluative measure of functional health that includes eight domain scores (general health, physical functioning, physical role performance, social functioning, emotional role performance, mental health, pain, vitality) that are summarized with the Physical Component Score (PCS) and Mental Component Score (MCS). The norm-based scoring system of the SF-36™ ranges from 0 to 100, with a normative mean of 50 and standard deviation of 10. Higher scores indicate better functional health.

**Information about relapse.**In the clinical trial, time to relapse was the primary endpoint. The Opticospinal Impairment Score (OSIS) [34] evaluated relapse severity in four domains: Visual Acuity, Motor Function, Sensory Function, and Sphincter Function. Scores range from 0 to 8 for the first domain, and 0 to 5 for all others. High scores indicate worse functioning.

On-trial relapse was defined as a patient’s new onset of neurologic symptoms or worsening of new neurologic symptoms if those symptoms persisted for more than 24 h, were attributed to NMO, and were preceded by at least 30 days of clinical stability. On the basis of the neurological exam and the OSIS, the treating clinician and a blinded examining clinician judged the severity of the relapse (“Clinician-Assessed Relapse”). A ‘major’ relapse was defined as an increase in 2–3 points in OSIS Visual Acuity (depending on whether the patient started with a score of 2–7 or 0–1, respectively); and an increase of 2–3 points on the OSIS Motor Subscale (depending on whether the patient started with a score of 2–6 or 0–1, respectively). Any loss in proprioception on the OSIS Sensory Subscale was considered ‘major.’

An independent panel of three experts (two neurologists and one neuroophthalmologist) who were blinded to treatment assignment then adjudicated the relapse by considering information from the Clinician-Assessed Relapse, Magnetic Resonance Imaging data, Optical Coherence Tomography imaging data, and the recorded exam. This adjudication process was intended to strengthen the robustness of the trial’s primary endpoint by reducing error variance due to (a) geographic differences in standards of care; and (b) a potential bias toward over-reporting a neurologic event as an on-trial relapse to mitigate potential long-term sequelae of a missed relapse. There were 43 patients with Clinician-Assessed Relapses, of whom 22 were adjudicated positively (i.e., categorized in the Adjudicated-Relapse group).

### Statistical analysis

This secondary analysis of NMOSD trial data examined evidence of response-shift effects in trial outcomes. We began by focusing on differences by treatment arm (Eculizumab vs. Placebo) and then examined differences by relapse status. Relapse status was defined as a three-level variable (No Relapse, Clinician-Assessed Relapse, Adjudicated Relapse). This variable allowed us to test relationships that had more power (due to larger sample size than simply comparing the Adjudicated-Relapse and No-Relapse groups), and that differentiated more subjective indicators of signal (i.e., Clinician-Assessed) from more objective indicators (i.e., Adjudicated).

These analyses aimed to “de-construct” different aspects of measurement invariance in the context of a small sample to characterize recalibration, reprioritization, and reconceptualization response shifts. We proceeded in four steps.

Step 1: Hypothesis-driven group differences in expected–observed discrepancy scores. This step tested the ‘omnibus response-shift hypothesis’ that there are differences between expected and observed QOL scores (“discrepancy scores”) as a function of the hypothesized response-shift catalyst (i.e., treatment arm and then whether the person ultimately had a relapse). If this omnibus test does not support a response-shift effect, then then subsequent analytic steps would not be implemented.
  To examine discrepancy-score differences by catalyst group, we used the Rapkin and Schwartz residual-modeling approach [17, 47]. We began by computing a principal component from the PRO scores, including the EQ-5D VAS and the 8 domain scores of the SF-36™ at all time points (Supplementary Table 1). This analysis enabled summarizing the PRO scores in one component score using data from all time points (see Results section for details).^1^ If this analysis had not supported the existence of one dominant component, we would have reduced the scores included such that they were well represented by a unidimensional component score. We then used this component score as the dependent variable in a random-effects model [48] that included the following demographic and clinical predictors at all available time points: gender, race, country, ethnicity, age, number of years since diagnosis, number of years since NMOSD-presenting symptoms, body mass index, and treatment compliance; and scores on the MRS, HAI, EDSS, and KFS. We saved the residuals from this model, and then tested models predicting these residuals (i.e., scores capturing the discrepancy between expected and observed outcomes) using hypothesized response-shift catalyst groups as the independent variable. Paneled histograms illustrate catalyst-group differences in the discrepancy scores.
  If these results suggested that there were response-shift effects, steps 2–4 would then examine evidence of recalibration, reprioritization, and reconceptualization response shifts, respectively. Random-effects models [48], and more specifically random-intercept models, were used to examine longitudinal differences in patterns of emphasis by catalyst group—whether PCS and MCS differed by treatment arm or relapse group in their ability to explain EQ-5D VAS scores; and whether such dynamics changed over time. (The decision to work with SF-36™ QOL summary [component] scores rather than domain scores was based on the statistical collinearity of the latter [e.g., average r_baseline_ = 0.40, range = 0.14–0.66], whereas the PCS and MCS scoring algorithm results in uncorrelated component scores [r_baseline_ =  − 0.13]).
Step 2: Group differences in patterns of emphasis. This analysis focused on characterizing a *recalibration response shift*. It examined whether the catalyst groups evinced different patterns of emphasis entailing a different connection between PCS or MCS and the EQ-5D VAS. This pattern would be indicated by significant two-way interactions (catalyst group-by-PCS; catalyst group-by-MCS) in a random-effects model predicting the EQ-5D VAS (dependent variable) from MCS, PCS, catalyst group, and Time (Weeks in Study). It is similar to asking in an Oort SEM [39] context whether the intercept of the slope relating the QOL component with the global ED-5D VAS is different by catalyst group.
Step 3: Group differences *over Time* in patterns of emphasis. This analysis focused on characterizing *reprioritization response shift*. Step 3 expanded on the prior model to investigate whether these patterns of emphasis changed differently by catalyst group *over time*. It tested the three-way interactions among catalyst group, Weeks in Trial, and PCS or MCS scores, after adjusting for main effects and the other interactions.
Step 4: We then tested for *reconceptualization response shift* by implementing a series of random-effects models predicting each QOL domain from catalyst group after adjusting for the other eight domains. Step 4 examined whether certain measures captured unique aspects of QOL that distinguished catalyst groups. This analysis focused on characterizing how each QOL domain’s relationship with catalyst-group status varied when isolated from (i.e., after adjusting for) the other QOL domains. We can infer reconceptualization response shift from this analysis based on how much catalyst-group variance is uniquely accounted for by each SF-36™ domain.

### Handling of missing data

There was very little missing data in this data set, and the variables we used in our modeling had no missing data.

## Results

### Sample

The study sample included 143 people, of whom 107 had Definitive Neuromyelitis Optica and 36 had NMO Spectrum Disorder (Table 1). Two-thirds of the sample was on Eculizumab and one-third on placebo, and the sample evinced high levels of treatment adherence. The sample had a mean age of 44 and a mean age of diagnosis of 41. The sample was predominantly female. Each patient had between three and 23 clinician visits during the trial, and each spent between two and 30 months under study.

**Table 1:** Descriptive statistics of study sample (*N* = 143 patients)

Variable	Mean	SD
Age	44.2	13.3
Range	19–75	
Age at NMO diagnosis	40.8	14.1
Range	14–73	
Body mass index	25.7	6.5
Range	15.4–49.0	
Treatment duration (Days)	566	400
Range	22	1478
Treatment compliance		
70–79%	1	1%
80–89%	12	8%
90–99%	50	35%
100%	80	56%

	*N*	%
Study arm		
Eculizumab	96	67%
Had an On-Trial Relapse	14	15%
Had an Adjudicated On-Trial Relapse	3	3%
Placebo	47	33%
Had an On-Trial Relapse	29	62%
Had an Adjudicated On-Trial Relapse	19	40%
NMO Diagnosis		
Definitive Neuromyelitis Optica	107	75%
NMO Spectrum Disorder	36	25%
Country		
USA	38	27%
Japan	14	10%
Rep. of Korea	13	9%
Russia	13	9%
Turkey	11	8%
Italy	7	5%
Thailand	7	5%
Argentina	6	4%
Germany	6	4%
Other (< 5 in each country)	28	20%
Gender		
Male	13	9%
Female	130	91%
Race		
American Indian or Alaskan Native	1	1%
Asian	52	36%
Black or African-American	17	12%
Other	1	1%
White	70	49%
Missing	2	1%

*NMO* Neuromyelitis Optica, *SD* standard deviation

**Fig. 2:**
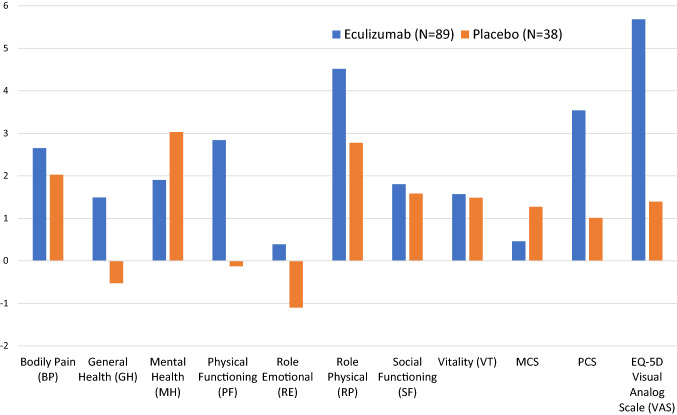
Mean change from baseline to study end in SF-36™ domain scores and EQ-5D VAS by treatment arm. The Eculizumab group evidenced bigger Changes in the SF-36™ physical domains compared to the Placebo group, which showed larger changes in the mental domains

Table 2 displays the descriptive statistics of baseline scores on the clinician- and patient-reported outcomes. On average, the sample had ‘slight disability’ on the MRS, and scores on the HAI and EDSS consistent with some gait abnormalities, but not enough to prevent independent walking. The sample’s average SF-36™ PCS score was substantially below norm-based means; the MCS score was slightly but not significantly below norm-based means. The biggest decrements on the SF-36™ domain scores were in physical functioning and physical role performance. On the EQ-5D VAS, mean scores reflected substantial health impairment. The Self-Care domain of the EQ-5D evinced the greatest decrement. Figure 2 shows the mean change from baseline on the SF-36™ domains by treatment arm. The Eculizumab group evidenced bigger changes in the SF-36™ physical domains compared to the Placebo group, which showed larger changes in the mental domains.

**Table 2:** Descriptive statistics of scores at baseline (*N* = 143)

Reported by	Item or (Sub)scale	Mean	SD	Min	Max
Clinician	Modified Rankin Score (MRS)	2.15	1.09	0	4
	Hauser Ambulation Index (HAI)	2.30	1.95	0	8
	Expanded Disability Status Score (EDSS)	4.18	1.60	1	7
Patient	SF-36 Physical Component Score (PCS)	38.02	10.17	8.45	65.56
	SF-36 Mental Component Score (MCS)	46.04	12.23	7.17	70.29
	Bodily Pain (BP)	42.12	10.97	21.68	62.00
	General Health (GH)	40.27	9.00	21.33	60.32
	Mental Health (MH)	45.48	11.50	11.63	63.95
	Physical Functioning (PF)	38.63	11.09	19.26	57.54
	Role Emotional (RE)	42.22	13.73	14.39	56.17
	Role Physical (RP)	36.81	11.29	21.23	57.16
	Social Functioning (SF)	41.42	11.68	17.23	57.34
	Vitality (VT)	44.75	10.02	22.89	67.45
	EQ-5D Visual Analogue Scale (VAS)	62.13	20.17	0	100

Where applicable, reported scores are norm-based rather than transformed

*SD* = standard deviation

### Component score used for creating discrepancy scores

**Fig. 3:**
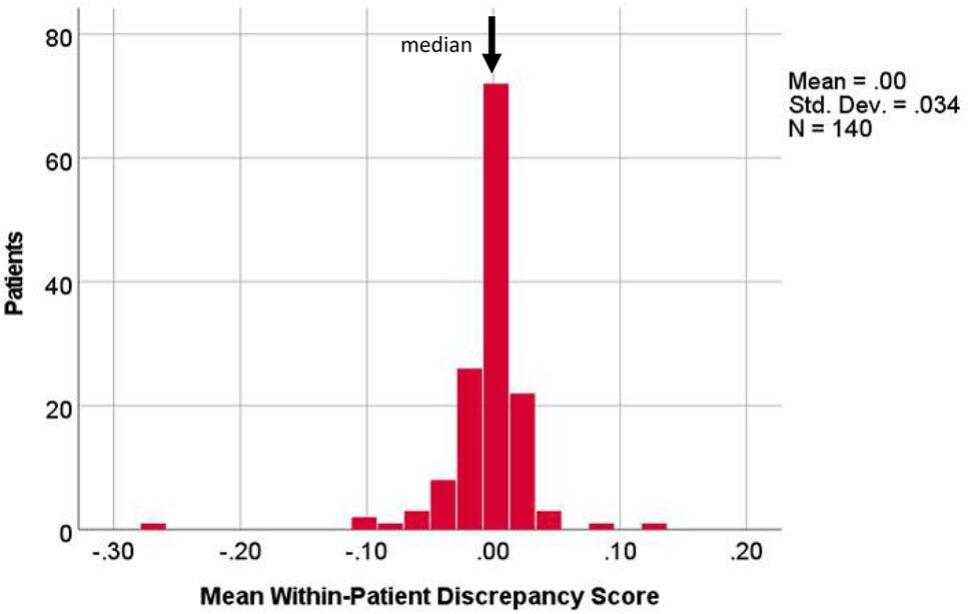
Discrepancy Score Distributions for the whole sample. The distribution was centered around zero, and slightly left-skewed

Supplementary Table 1 shows the loadings of each of the PROs used in the PCA. The PRO data from all time points were effectively captured in one component score (Successive Eigenvalues = 4.95, 0.96, and 0.85; successive variances explained = 55%, 10.7%, and 9.4%.). Fig. 3 shows the distribution of the discrepancy scores in the entire sample. The distribution was centered around zero, and slightly left-skewed.

### Treatment arm as catalyst

#### Step 1: Treatment arm differences in expected-versus-observed discrepancy scores

**Fig. 4:**
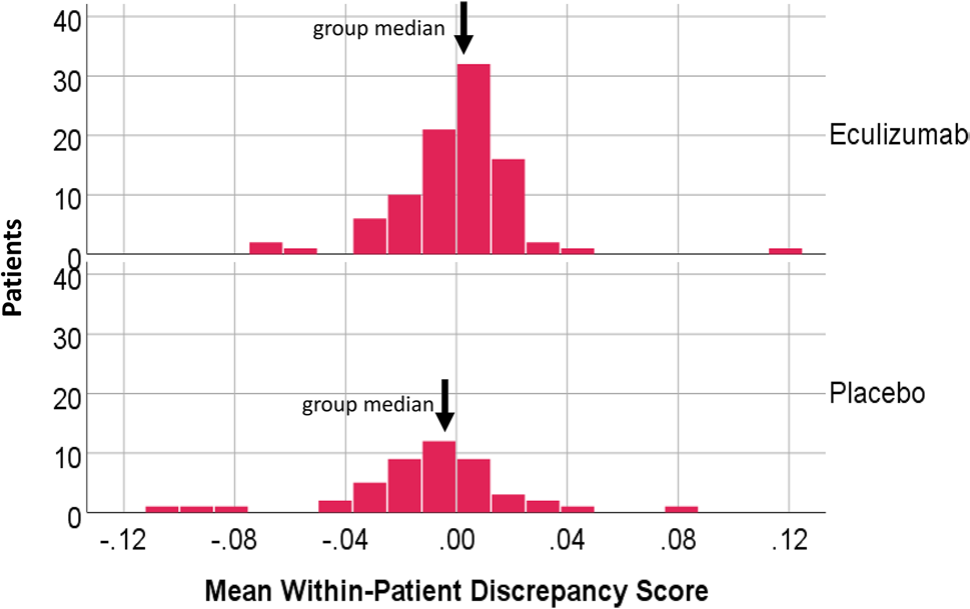
Discrepancy Score Distributions by Treatment Arm. The distributions of discrepancy scores are different by treatment arm, with the largest and more-negative discrepancies found in the Placebo group, as compared to the Eculizumab group. One low-scoring outlier was not shown in the graph but was retained in calculations

The Kruskal–Wallis non-parametric test revealed differences in the central tendencies of the distributions of the discrepancy score by treatment arm (test statistic = 108.40, df = 1, *p* < 0.0005). The placebo group had a systematically lower median (Fig. 4). For the Eculizumab patients, the discrepancy score was generally close to zero. A sensitivity test was done omitting one low-scoring outlier in the Placebo group and the results were essentially unchanged.

#### Step 2: Treatment arm differences in patterns of emphasis

Table 3 shows the results of random-effects models assessing differences in patterns of emphasis in the trial participants. There were significant two-way interactions between treatment arm and PCS and MCS scores, such that the Placebo patients had a greater emphasis on PCS and a lesser emphasis on MCS in their ED-5D VAS scores as compared to the Eculizumab patients.

**Table 3:** Random effects models testing treatment-arm-related differences in emphasis

Type III tests of fixed effects: dependent variable: EQ-5D VAS score				
Source	Numerator df	Denominator df	*F*	Sig
Intercept	1	900.66	0.87	0.35
Weeks	1	253.53	1.52	0.22
Treatment Arm	1	900.66	1.57	0.21
SF-36 PCS	1	966.50	138.63	**0.00**
SF-36 MCS	1	1295.42	100.60	**0.00**
Treatment Arm * Weeks	1	253.53	0.57	0.45
Weeks * SF-36 PCS	1	248.21	0.80	0.37
Weeks * SF-36 MCS	1	467.63	0.14	0.71
Treatment Arm * SF-36 PCS	1	966.50	8.63	**0.00**
Treatment Arm * SF-36 MCS	1	1295.42	4.45	**0.04**
Treatment Arm * Weeks * SF-36 PCS	1	248.21	2.21	0.14
Treatment Arm * Weeks * SF-36 MCS	1	467.63	0.62	0.43

Estimates of fixed effects: dependent variable: EQ VAS score							
Parameter	Estimate	SE	df	*t*	Sig	95% confidence interval	
						Lower bound	Upper bound
Main effects							
Intercept	9.16	4.79	813.89	1.91	*0.06*	− 0.24	18.57
Weeks	0.03	0.06	124.78	0.52	0.61	− 0.09	0.16
Treatment Arm = Placebo	− 10.50	8.37	900.66	− 1.25	0.21	− 26.93	5.94
Treatment Arm = Eculizumab	*Referent*	0.00					
SF-36 PCS	*0.64*	0.08	1005.57	8.03	**0.00**	0.49	0.80
SF-36 MCS	0.66	0.06	1057.45	11.08	**0.00**	0.55	0.78
Two-way interactions							
Treatment Arm = Placebo * Weeks	0.11	0.14	253.53	0.76	0.45	− 0.17	0.38
Treatment Arm = Eculizumab * Weeks	*Referent*	0.00					
Weeks * SF-36 PCS	0.00	0.00	125.45	0.64	0.52	0.00	0.00
Weeks * SF-36 MCS	0.00	0.00	288.19	− 1.25	0.21	0.00	0.00
Treatment Arm = Placebo * SF-36 PCS	*0.43*	0.15	966.50	2.94	**0.00**	0.14	0.71
Treatment Arm = Eculizumab * SF-36 PCS	*Referent*	0.00					
Treatment Arm = Placebo * SF-36 MCS	− 0.23	0.11	1295.42	− 2.11	**0.04**	− 0.45	− 0.02
Treatment Arm = Eculizumab * SF-36 MCS	*Referent*	0.00					
Three-way interactions							
Treatment Arm = Placebo * Weeks * SF-36 PCS	*0.00*	0.00	248.21	− 1.49	0.14	− 0.01	0.00
Treatment Arm = Eculizumab * Weeks * SF-36 PCS	*Referent*	0.00					
Treatment Arm = Placebo * Weeks * SF-36 MCS	*0.00*	0.00	467.63	0.79	0.43	0.00	0.00
Treatment Arm = Eculizumab * Weeks * SF-36 MCS	*Referent*	0.00					

Bolded significance values have p < 0.05. Italicized significance values have p < 0.10

*PCS* = physical component score, *MCS* =  mental component score

#### Step 3: Treatment arm differences in changes over time in patterns of emphasis

There were no significant three-way interactions for Treatment Arm with time and PCS or MCS (Table 3). These results suggest that differences in patterns of emphasis did not change over time. Residuals overall and for each group were non-normal (*p* < 0.0005 for each treatment arm) due to skewness (− 0.57 and − 0.39, for Placebo and eculizumab, respectively).

#### Step 4: Group differences in conceptualization of QOL

Table 4 shows results of the series of random-effects models aimed at clarifying how each domain’s relationship with Treatment Arm varied across models when adjusting for all the other domains. These models suggested that the Placebo group was associated with substantially worse-than-expected ED-5D VAS and Vitality scores. None of the other seven SF-36™ domain scores had statistically important relationships with Treatment Arm after adjusting for the other QOL domain scores.

**Table 4:** Isolating QOL associations by treatment arm

	Placebo
EQ-5D VAS	** − 4.0**+
SF-36™ Bodily Pain	** − **1.7
SF-36™ General Health	0.4
SF-36™ Mental Health	1.3
SF-36™ Physical Function	− 1.2
SF-36™ Role Emotional	− 0.3
SF-36™ Role Physical	− 0.5
SF-36™ Social Function	0.5
SF-36™ Vitality	− 3.1***

*SF-36=*Short-Form 36, *EQ-5D VAS* European quality of life 5-dimension visual analogue scale

+ *p *= 0.07

***p < 0.0001

Because Eculizumab was highly effective at preventing relapse, we hypothesized that the response-shift effects related to treatment arm overwhelmingly reflected the impact of relapse on patients. We thus investigated response-shift effects by relapse status using the same series of analyses.

### Relapse group as catalyst

#### Step 1: Relapse-group differences in expected-versus-observed discrepancy scores

**Fig. 5:**
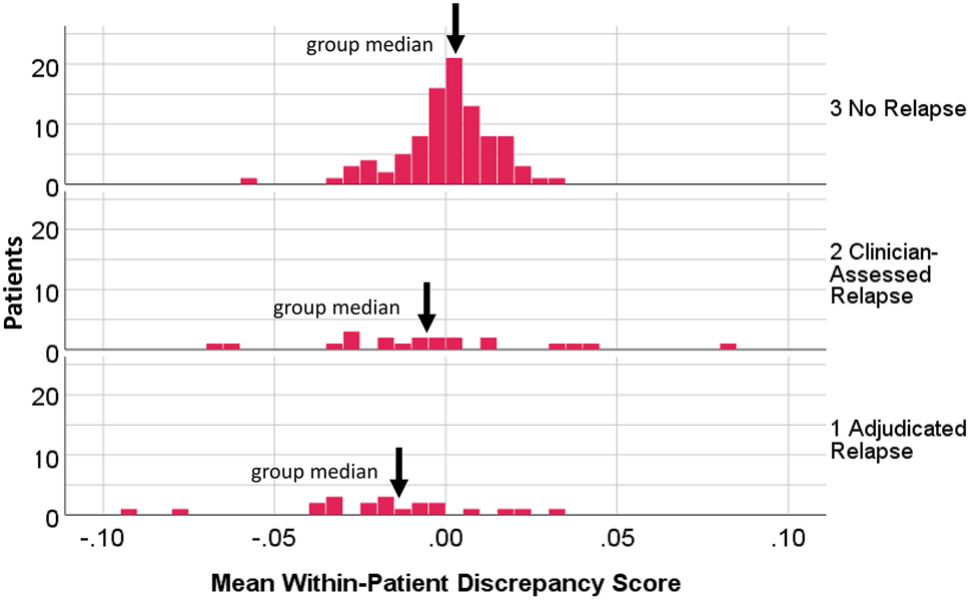
Discrepancy Score Distributions by Relapse Group. The distributions of discrepancy scores are different by relapse group, with the largest and more-negative discrepancies found in the Adjudicated-Relapse as compared to the Clinician-Assessed and No-Relapse Groups. One low-scoring outlier was not shown in the graph but was retained in calculations

The Kruskal–Wallis non-parametric test supported that there were relapse-group differences in the discrepancy-score distributions (test statistic = 14.87, df = 2, *p* = 0.001). Figure 5 shows the distribution of discrepancy scores by relapse group. For No-Relapse patients, the discrepancy score was generally close to zero. For the Clinician-Assessed Relapse and Adjudicated-Relapse groups, the score varied more widely. Post hoc pairwise comparisons revealed that the Adjudicated-Relapse group had substantially larger and more-negative discrepancy scores than the other groups (K-W Test Statistics =  − 35.99 versus − 14.97 and − 21.02, respectively; *p* < 0.0001 versus *p* = 0.125 and 0.09, respectively). A sensitivity test omitted the low-scoring outlier in the No-Relapse group and the results were essentially unchanged.

#### Step 2: Relapse-group differences in patterns of emphasis

Table 4 shows results of random-effects models assessing differences in patterns of emphasis in the trial participants. There was a significant two-way interaction between Adjudicated Relapse and PCS, and Clinician-Assessed Relapse and MCS. Thus, compared to the No-Relapse patients, Adjudicated-Relapse patients had a greater emphasis on PCS in their ED-5D VAS scores, and Clinician-Assessed relapse patients had a greater emphasis on MCS in their ED-5D VAS scores.

#### Step 3: Relapse-group differences in changes over time in patterns of emphasis

There were significant three-way interactions for Relapse-by-time-by-PCS and Relapse-by-time-by-MCS (*b* =  − 0.01 in both cases; *p* = 0.02 and 0.01, respectively), after adjusting for main effects and two-way interactions (Table 5). These results suggest that although PCS and MCS are more important in accounting for ED-5D VAS for people who had an adjudicated relapse than for people with no relapse, this difference attenuates over time. Residuals overall and especially for the no-relapse group were non-normal (*p* < 0.0005 and 0.0005, respectively) due to skewness (− 0.48 and − 0.52, respectively). For the adjudicated and clinician-assessed relapse, the residuals were normally distributed (*p* = 0.05 and 0.50, respectively).

**Table 5:** Random effects models testing relapse-related differences in emphasis by relapse group and over time

Type III tests of fixed effects: dependent variable: EQ-5D VAS score				
Source	Numerator df	Denominator df	*F*	Sig
Intercept	1	702.74	2.17	0.14
Weeks	1	643.17	14.34	**0.00**
Relapse (Three-level variable)	2	719.81	5.92	**0.00**
SF-36 PCS	1	688.01	89.58	**0.00**
SF-36 MCS	1	1177.91	116.32	**0.00**
Relapse * Weeks	2	598.15	7.98	**0.00**
Weeks * SF-36 PCS	1	573.96	5.40	**0.02**
Weeks * SF-36 MCS	1	345.09	7.54	**0.01**
Relapse * SF-36 PCS	2	687.40	2.92	*0.05*
Relapse * SF-36 MCS	2	1180.61	4.12	**0.02**
Relapse * Weeks * SF-36 PCS	2	535.78	3.14	**0.04**
Relapse * Weeks * SF-36 MCS	2	344.49	4.19	**0.02**

Estimates of fixed effects: dependent variable: EQ VAS score							
Parameter	Estimate	SE	df	*t*	Sig	95% confidence interval	
						Lower bound	Upper bound
Main effects							
Intercept	11.63	4.57	850.90	2.54	**0.01**	2.66	20.60
Weeks	0.02	0.06	121.34	0.25	0.80	− 0.10	0.14
Adjudicated relapse	− 36.01	11.46	896.94	− 3.14	**0.00**	− 58.50	− 13.53
Clinician-assessed relapse	− 22.38	12.00	585.46	− 1.86	*0.06*	− 45.95	1.19
No Relapse	*Referent*	0.00					
SF-36 PCS	0.77	0.08	1013.18	10.24	**0.00**	0.63	0.92
SF-36 MCS	0.51	0.06	1082.46	8.72	**0.00**	0.40	0.63
Two-way interactions							
Adjudicated Relapse * Weeks	0.99	0.26	469.55	3.88	**0.00**	0.49	1.49
Clinician-Assessed Relapse * Weeks	0.29	0.25	797.25	1.16	0.24	− 0.20	0.77
No Relapse * Weeks	*Referent*	0.00					
Weeks * SF-36 PCS	0.00	0.00	121.02	− 0.31	0.76	0.00	0.00
Weeks * SF-36 MCS	0.00	0.00	286.98	0.43	0.66	0.00	0.00
Adjudicated Relapse * SF-36 PCS	0.51	0.22	872.94	2.31	**0.02**	0.08	0.94
Clinician-Assessed Relapse * SF-36 PCS	− 0.09	0.20	569.25	− 0.42	0.67	− 0.49	0.32
No Relapse * SF-36 PCS	*Referent*	0.00					
Adjudicated Relapse * SF-36 MCS	0.22	0.14	1244.70	1.56	0.12	− 0.06	0.50
Clinician-Assessed Relapse * SF-36 MCS	0.41	0.15	1124.81	2.63	**0.01**	0.10	0.71
No Relapse * SF-36 MCS	*Referent*	0.00					
Three-way interactions							
Adjudicated Relapse * Weeks * SF-36 PCS	* − 0.01*	0.00	439.02	− 2.43	**0.02**	− 0.02	0.00
Clinician-Assessed Relapse * Weeks * SF-36 PCS	− 0.004	0.01	670.12	− 0.73	0.47	− 0.01	0.01
No Relapse * Weeks * SF-36 PCS	*Referent*	0.00					
Adjudicated Relapse * Weeks * SF-36 MCS	− 0.01	0.00	276.16	− 2.48	**0.01**	− 0.02	0.00
Clinician-Assessed Relapse * Weeks * SF-36 MCS	− 0.01	0.00	453.64	− 1.59	0.11	− 0.01	0.00
No Relapse * Weeks * SF-36 MCS	*Referent*	0.00					

Bolded significance values have *p* ≤ 0.05. Italicized significance values have *p* < 0.10

*SF-36* = Short-Form 36, *PCS* = physical component score, *MCS* = mental component score, *EQ-5D VAS* = European Quality of Life 5-Dimension Visual Analogue Scale

#### Step 4: Relapse-group differences in conceptualization of QOL

Table 6 shows results of the series of random-effects models aimed at clarifying how each domain’s relationship with relapse status varied across models when adjusting for all the other domains. These models suggested that relapse status was associated with substantially worse-than-expected ED-5D VAS scores for both Clinician-Assessed and Adjudicated-Relapse groups, after adjusting for the 8 SF-36™ domain scores. In other words, in contrast to the SF-36™ domain scores, ED-5D VAS scores uniquely discriminated Relapse-Group deficits. On the other hand, people who had a Clinician-Assessed Relapse had slightly better than expected Social-Function scores. In other words, Social-Function scores uniquely revealed a strength of this Group. None of the other seven SF-36™ domain scores had statistically important relationships with relapse status after adjusting for the other QOL domain scores.

**Table 6:** Isolating QOL associations with relapse status

Dependent variable	Parameter estimate^†^	
	Clinician-assessed relapse	Adjudicated relapse
EQ-5D VAS	− 7.8*	− 6.5*
SF-36™ Bodily Pain	− 1.7	− 2.1
SF-36™ General Health	− 2.1	− 0.1
SF-36™ Mental Health	− 0.7	− 0.2
SF-36™ Physical Function	− 2.4	0.5
SF-36™ Role Emotional	− 1.7	− 2.1
SF-36™ Role Physical	− 0.7	− 1.7
SF-36™ Social Function	2.8*	0.1
SF-36™ Vitality	0.8	− 1.7

*SF-36* Short-Form 36

*p < 0.05

^†^Parameter estimates are not shown for individual SF-36™ domains that are covariates in a given model

## Discussion

This secondary analysis of clinical trial data revealed that not receiving active treatment and, more specifically, the experience of relapse made people change their thinking about QOL (see summary in Table 7). The implications for such changes on interpreting treatment effects may be substantial. Our results suggest that the QOL impacts of placebo/relapse were under-estimated by the usual analyses, and thus the benefit of Eculizumab is likely even greater than what was documented in the pivotal clinical trial [33], extending to subjective outcomes.

**Table 7:** Summary of response-shift analyses and interpretation

Response-shift evidence	Step #	Research question	Statistical support; table or figure	Interpretation of finding(s)
Omnibus evidence of response shift?	1	Are there differences in discrepancy scores by Relapse Group?	Kruskal–Wallis test comparing discrepancy distributions by Treatment Arm and Relapse Group; Figs. 2 and 3	The ‘standard QOL model’ (i.e., one that includes demographic and objective indicators of neurologic functioning) reflects substantially less well the experience of being on Placebo and of having had a relapse as compared to being on Eculizumab or having not had a relapse.
Recalibration response shift?	2	Is there evidence of Relapse Group-related differences in patterns of emphasis (i.e., connection between VAS and PCS and/or MCS scores)?	2-way interactions: Treatment Arm*PCS, Treatment Arm*MCS; and Relapse Group*PCS, Relapse Group*MCS; Tables 3 and 5	Placebo patients had a greater emphasis on PCS and lesser emphasis on MCS in their VAS scores, as compared to Eculizumab patients. Clinician-Assessed relapse patients had a greater emphasis on MCS in their VAS scores as compared to the No-Relapse patients. Adjudicated-Relapse patients showed a greater emphasis on PCS than the No-Relapse patients.
Reprioritization response shift?	3	Are there Relapse Group-related differences in changes in emphasis over time?	3-way interactions: None for Treatment Group; Relapse Group*Weeks*PCS, Relapse Group*Weeks*MCS; Table 3	There was no differential effect over time in patterns of emphasis by treatment arm. Over time, however, the patterns of emphasis differed across Relapse Groups. The importance of PCS and MCS for Adjudicated patients attenuated over time.
Reconceptualization response shift?	4	Do certain measures capture unique aspects of QOL that distinguish Relapse Groups?	Series of regressions to isolate unique contributions of QOL domain scores; Table 4	In contrast to the SF-36™ domain scores, VAS scores uniquely discriminated Relapse-Group deficits. Social-Function scores uniquely revealed a strength of the Clinician-Assessed Relapse Group.

*QOL* = quality of life, *PCS* = physical component score, *MCS* = mental component score

Of note, the whole study sample started the trial with close-to-normal scores on the MCS, despite decidedly low scores on the PCS and ED-5D VAS. Thus, despite having dealt with the vicissitudes of NMOSD for an average of 4 years, the participants managed to maintain prior to the trial a relatively normal level of mental-health functioning. In this they also managed to maintain stability over the course of the trial, regardless of treatment arm. This paradox is consistent with response-shift theory, which posits that changes in internal standards, values, and conceptualizations of health allow individuals to maintain QOL homeostasis in the face of changing health circumstances [2, 17].

Our findings likely reflect the ‘shadow’ of response shift, inferred by the behavior of examined interactions and unique variance explained rather than characterized more directly. People on placebo and/or people who had a relapse are thinking differently about health due to their experiences. The relapse experience appears to reflect less and less that which is assessed by the SF-36™ generic functional health indicators, and so assessment of more constructs would be required to delineate exactly what ‘health’ means after relapse. For example, ‘health’ may have more to do with purpose in life or meaningful social connections, concepts measured by the Ryff Psychological Well-Being scale [49, 50]. Including measures of cognitive appraisal [51, 52] would also facilitate more direct characterization of the response-shift effects. Nevertheless, in the absence of other such measures, the ED-5D VAS has clear value in this study.

The present study represents a response-shift investigation of clinical trial data using accepted analytic methods. Triggered by prior unexpected non-significant treatment differences in the more subjective domains related to mental health, here we pursued a series of analyses to explicate these patterns. These analytic steps begin by testing an omnibus response-shift hypothesis that examines the distribution of discrepancy scores by catalyst group. If this hypothesis does not support response shift, then no further analyses would be done. In our companion paper [53], we provide a method that builds on these findings to enable estimation of how response shift affects measured outcomes.

It should be noted that the residual-modeling approach specified in our analyses is distinct from Mayo’s 2008 method [7]. While Mayo’s 2008 method also works with residuals, the Rapkin and Schwartz method [17] explicitly computes a ‘standard model’ that includes all available antecedents, and saves the residuals (i.e., discrepancies), which are then used as the dependent variable in hypothesis-driven analyses. Once the response-shift omnibus hypothesis is supported (i.e., the aforementioned discrepancies differ by catalyst group), the method presented in this article then implements a series of random-effects models to test response-shift effects operationalized in ways similar to the Oort SEM method. If measures of appraisal had been collected in the trial data, the Rapkin and Schwartz method would also examine main effects and interactions of appraisal and change in appraisal in conjunction with catalyst (i.e., treatment arm or relapse status) main effects and interactions. In contrast, the Mayo method creates residuals based on a short list of antecedents (i.e., disease severity, age, sex, and comorbidity), and then creates residual-trajectory scores which are then modeled using latent class analysis. Both methods utilize residuals to test response-shift hypotheses in interesting and informative ways, but the method used in our work is correctly identified as the Rapkin and Schwartz (2004) method [17].

The study has a number of strengths including the high-quality data on relapse, the inclusion of subjective and objective indicators, and the longitudinal follow-up with low attrition. Its limitations must, however, be acknowledged. Our results likely under-estimate response-shift effects for several reasons. First, the sample sizes of those who ultimately had an adjudicated relapse are relatively small, affording statistical power to detect only large effect sizes [54]. Accordingly, some models may be over-identified. To reach significance despite low power means more than to do so when aided by high power. This situation prevents the application of well-codified response-shift analyses using SEM that would enable us to work with collinear domain scores (using residual correlation), and to model moderation and mediation effects more robustly. The residuals from the two random-effects models were also not always normally distributed, which violates a random-effects model assumption [48, 55]. Random-effects models appear, however, to be robust to such violations [56, 57]. Further, the study does not include measures of certain relevant constructs such as well-being or of cognitive processes underlying patient self-report. Measures of QOL appraisal processes [51, 52] would facilitate a more narrative and nuanced description of how the relapse groups differed in their frames of reference, standards of comparison, experience sampling, and patterns of emphasis [58, 59]. Future research might include such cognitive-appraisal and well-being scales [49, 50] in prospective clinical trials of new treatments to ensure that the patients’ experience is captured over the course of the trial. Finally, most data that were collected from those who ultimately suffered a relapse were collected *before* that relapse. Thus, the study design afforded little opportunity for detecting Relapse-Group differences. Despite these odds, we found such differences, perhaps suggesting that relapse patients are experiencing sub-clinical, early warning signs of a relapse. Further investigation into these early warning signs might enable interventions to delay the ‘tipping point’ to full relapse [60, 61].

In summary, this study of response-shift effects in the Eculizumab clinical trial suggests response-shift effects by treatment arm and relapse status. Using a series of analytic steps aimed at detecting the ‘shadow’ or reflection of response shift, we found that, among Placebo patients and as relapse criteria became more specific and rigorous, commonly accepted clinical and demographic indicators explained less well the patients’ QOL ratings. The idea of ‘health’ among placebo patients and among those who eventually relapsed reflected different patterns of emphasis, and these emphases changed over time for relapse patients, compared to the No-Relapse Group, even when these differences were “watered down” by the inclusion of pre-relapse data. We conclude that there are other aspects of QOL that become more important when one experiences a relapse, aspects that are not well captured in the SF-36™ and/or EQ-5D VAS. This ‘shadow’ of response shift may take a more definite shape when more relevant constructs are included in a study well powered to explicate the relapse experience.

## Electronic supplementary material

Below is the link to the electronic supplementary material.

Electronic supplementary material 1 (PDF 85 kb)

## Data Availability

The study data are confidential and thus not able to be shared.

## Abbreviations

MCS: Norm-based mental component score of the SF-36™
NMOSD: Neuromyelitis optica spectrum disorder
OSIS: Opticospinal impairment score
PCS: Norm-based physical component score of the SF-36™
PRO: Patient-reported outcome
SF-36v2™: Short-form-36v2
QOL: Quality of life
VAS: Visual-Analogue-Scale indicator of global health on the European Quality of Life 5-Dimension (EQ-5D

## Footnotes

1 The use of a PCA to summarize PROs for use in computing discrepancy scores can easily be modified to include only one PRO if that were in fact the available data.
