# Supplementary material for: Response-shift effects in neuromyelitis optica spectrum disorder: a secondary analysis of clinical trial data
Source: Qual Life Res. 2020 Dec 2;30(5):1267–82. doi: 10.1007/s11136-020-02707-y (PMC8068626; doi:10.1007/s11136-020-02707-y)
Supplement: Supplementary file 1 — Electronic supplementary material 1 (PDF 85 kb) [file 11136_2020_2707_MOESM1_ESM.pdf]

Supplemental Table 1. Results of Principal Components Matrix of QOL Domain Scores†*

	Loading	
EQ-5D VAS Score	0.74	
SF-36™ Bodily Pain	0.71	
SF-36™ General Health	0.69	
SF-36™ Mental Health	0.70	
SF-36™ Physical Functioning	0.68	
SF-36™ Role Emotional	0.74	
SF-36™ Role Physical	0.82	
SF-36™ Social Functioning	0.82	
SF-36™ Vitality	0.76	
Eigenvalue		4.95
PCA explained variance		55%

† All SF-36™ scores are norm-based.

**All time points used in analysis.*
